# The cGAS/STING–TBK1–IRF Regulatory Axis Orchestrates a Primitive Interferon-Like Antiviral Mechanism in Oyster

**DOI:** 10.3389/fimmu.2021.689783

**Published:** 2021-06-08

**Authors:** Xue Qiao, Yanan Zong, Zhaoqun Liu, Zhaojun Wu, Yuanmei Li, Lingling Wang, Linsheng Song

**Affiliations:** ^1^ Liaoning Key Laboratory of Marine Animal Immunology, Dalian Ocean University, Dalian, China; ^2^ Functional Laboratory of Marine Fisheries Science and Food Production Processes, Qingdao National Laboratory for Marine Science and Technology, Qingdao, China; ^3^ Liaoning Key Laboratory of Marine Animal Immunology and Disease Control, Dalian Ocean University, Dalian, China; ^4^ Southern Laboratory of Ocean Science and Engineering (Guangdong, Zhuhai), Zhuhai, China; ^5^ Dalian Key Laboratory of Aquatic Animal Disease Prevention and Control, Dalian Ocean University, Dalian, China

**Keywords:** *Crassostrea gigas*, interferon-like system, cyclic GMP-AMP (cGAMP) synthase (cGAS), stimulator of interferon genes (STING), antiviral immunity

## Abstract

Interferon (IFN) system is considered as the first defense line against viral infection, and it has been extensively studied in vertebrates from fish to mammals. In invertebrates, Vagos from arthropod and IFN-like protein (*Cg*IFNLP) from *Crassostrea gigas* appeared to function as IFN-like antiviral cytokines. In the present study, the *Cg*IFNLP protein in hemocytes was observed to increase after Poly (I:C) stimulation. After *Cg*IFNLP was knocked down by RNAi, the mRNA expression of IFN-stimulated genes (*Cg*ISGs) was significantly inhibited. Both cyclic GMP-AMP synthase (*Cg*cGAS) and stimulator of interferon gene (*Cg*STING) identified from oyster were able to recognize the double-stranded nucleic acid [Poly (I:C) and dsDNA] and expressed at high level after Poly (I:C) stimulation. The expression of *Cg*IFNLP and interferon regulatory factors (*Cg*IRF1/8) and the nuclear translocation of *Cg*IRF8 were all suppressed in *Cg*cGAS-RNAi or *Cg*STING-RNAi oysters after Poly (I:C) stimulation. The expression level of *Cg*STING and TANK binding kinase1 (*Cg*TBK1) did not decrease in *Cg*cGAS-RNAi oysters. After *Cg*STING was knocked down, the high expression of *Cg*TBK1 induced by Poly (I:C) was prevented significantly. These results indicated that there was a primitive IFN-like antiviral mechanism dependent on the cGAS/STING–TBK1–IRFs regulatory axis in mollusks, which was different from the classic cGAS–STING–TBK1 signal pathway in mammals.

## Introduction

Interferon (IFN) system is the vital component of immune response in mammal, and it is recognized as the first line of defense against viral infection. IFNs don’t possess the direct antiviral activity, and they are so named because of the shared property to impede viral replication at numerous stages of the viral lifecycle, which is indeed critical for activating a robust host response against viral infection (1). The released IFNs engage their cognate receptors at the cell surface, then trigger JAK–STAT signaling and ultimately lead to the transcriptional activation of IFN-stimulated genes (ISGs) (2). The proteins encoded by ISGs, such as interferon-induced proteins, myxovirus resistance (Mx), and Viperin, can disrupt the life cycle of virus and reduce the damage caused by virus infections (3).

The signal pathways to mediate the expression of IFNs are relatively conservative, even though the IFNs are principally expressed by different kinds of innate immune cells in mammal (4). The reaction cascade to regulate the production of IFNs can be initiated by the specific binding of pathogen-associated molecular patterns (PAMPs) to pattern recognition receptors (PRRs), such as Toll like receptors (TLRs), retinoic acid-inducible gene I (RIG-I), melanoma differentiation associated protein 5 (MDA5), cyclic GMP-AMP (cGAMP) synthase (cGAS), and nucleotide binding oligomerization domain-like receptors (NLRs) (5–9). For instance, RIG-I/MDA5 specializes in discriminating pathogen-derived RNA (**e.g.**, dsRNA or 5′ppp-RNA) in the cytoplasm, while cGAS recognizes cytosolic DNA in a sequence-independent manner (10, 11). The activated cGAS catalyzes the reaction to generate 2′3′-cGAMP which then activates the stimulator of interferon genes (STING) (12). The STING recruits the kinases TANK binding kinase 1 (TBK1) and Ikappa B Kinase *Ɛ* (IKK*Ɛ*) to phosphorylate IFN regulatory factor IRF-3/7, which translocates into the nucleus to initiate the expression of IFNs (13). The IRF family members are renowned for their involvement in the regulation of IFN system (14). For instance, the IRF-3 and IRF-7 are essential for the regulated expression of IFNs, and IRF-9 is indispensable for gene transcription of ISGs by combining the STAT1 and STAT2 (15).

The IFN mediated antiviral response displays conserved property in fishes and all tetrapods, but not in tunicates or amphioxus (16). It has been suspected that the IFN system derives from earliest jawed vertebrates and evolves from a class II helical cytokine ancestor, along with the IL-10 cytokine family (17–19). Recently, the accumulating pieces of evidence have demonstrated that the nucleic acid-induced antiviral immunity also exists in invertebrates with the similar characteristic of IFN responses in mammals, even there is no identified IFN homologous (20). It is worth noting that Vago appears to function as an IFN-like antiviral cytokine in arthropods, such as *Dm*Vago in *Drosophila* (21), *Cx*Vago in *Culex* (22), and *Lv*Vago in *Litopenaeus vannamei* (23). These Vagos restrict virus infection by the activation of JAK–STAT pathway, indicating the existence of IFN-like system in arthropods. Meanwhile, many elements of IFN signaling pathway were recently discovered in abalone, oyster, and clam, which provided the evidence of functional homolog of IFN in mollusks. For instance, *Mx* and Viperin were identified in *Haliotis discus discus* and *C. gigas* respectively (24, 25), and several IRFs were found to participate in antiviral immune response in *Pinctada fucata* and *C. gigas* (26, 27).

The Pacific oyster *C. gigas* is the dominant farmed oyster species worldwide. In the past decades, the oyster aquaculture suffered from mass mortalities, and the *Ostreid herpesvirus* (OsHV-1) was considered to be one of the major virus pathogen (28, 29). In our previous works, an IFN-like protein (*Cg*IFNLP) and an IFN receptor-like 3 (*Cg*IFNR-3) were identified from *C. gigas* (30, 31), and their mRNA expression levels in hemocytes increased significantly after Poly (I:C) stimulation. *Cg*IFNR-3 interacted with *Cg*IFNLP *in vitro*, and it could activate the expression of human interferon-stimulated response element (ISRE), STAT3, and GAS in the reporter luciferase activity assay (31). In the present study, the cGAS and STING homologs (designated as *Cg*cGAS and *Cg*STING) were identified from *C. gigas* with the main objectives (1) to determine their recognition towards double-stranded nucleic acid and the effect on nuclear translocation of *Cg*IRFs, (2) to illuminate their regulatory mechanism for *Cg*IFNLP expression, (3) to examine the expression changes of *Cg*ISGs after *Cg*IFNLP was knocked down, and clarify the *Cg*cGAS/*Cg*STING mediated primitive IFN-like antiviral mechanism in oyster.

## Material and Methods

### Experimental Animals and Sample Collection

All experiments were performed in accordance with the approval and guidelines of the Ethics Review Committee of Dalian Ocean University. Adult Pacific oysters *C. gigas* (about 13.0 cm in shell length, average weight of 100 g) collected from a local farm in Dalian, Liaoning Province, China, were cultured in laboratory aquarium tanks with aerated seawater at 15–20°C for one week before processing. Six-week old Kunming mice were provided by the Experimental Animal Center at Dalian Medical University, Dalian, China and raised and handled under pathogen-free conditions.

One hundred and eight oysters were randomly divided into two groups. The oysters in Poly (I:C) stimulation group and control group received individually an injection with 0.1 ml of Poly (I:C) (1 mg/ml, dissolved in seawater) (Sigma-Aldrich, USA) and seawater, respectively. The hemocytes were collected from nine oysters in each group at 0, 3, 6, 12, 24, and 48 h after injection. The hemocytes from three oysters were pooled together as one sample, and there were three replicates for each time point. The hemocytes were harvested by centrifugation at 800 × *g*, 4°C for 8 min. Meanwhile, the tissues including gonad, adductor muscle, mantle, gills, hemolymphs, labial palps, and hepatopancreas were collected from untreated oysters.

### cDNA Cloning and Sequence Analysis

The full-length cDNA fragments of *Cg*cGAS, *Cg*STING, and *Cg*IRF-8 were obtained by PCR according to the previous report (31). The primers (**Table S1**, P1–6) were designed according to sequence information (XM_034454330.1, XM_011452302.3, XM_011414344.3) from the National Center for Biotechnology Information database (https://www.ncbi.nlm.nih.gov/). BLASTx (http://www.ncbi.nlm.nih.gov/) was used for homology analysis, and ExPASy (http://www.expasy.org/) was employed to predict the amino acid sequences. SMART (http://smart.embl-heidelberg.de/) was used to predict the protein domains. MEGA 6.0 program and Clustal X were used for phylogenetic analysis and multiple sequence alignment, respectively.

### RNA Extraction,cDNA Synthesis and Quantitative Real-Time *PCR* Analysis

Total RNA was isolated from all the tissue and hemocyte samples using Trizol reagent (Thermo Fisher Scientific, USA) and synthesized into cDNA with PrimeScript Reverse Transcriptase following the manufacturer’s introduction (Takara, China). The qRT-PCR was carried out according to the method described previously with the designed primers (**Table S1**, P7–P26) (32). Briefly, qRT-PCR was performed with the SYBR premix ExTap (RR420, Takara, Japan) on ABI PRISM 7500 Sequence Detection System (Thermo Fisher, USA). The relative expression level was calculated by 2^-ΔΔCt^ method with *Cg*EF (NM_001305313.2) as internal reference. Dissociation curve analysis of amplification products was performed to confirm the specificity of amplification.

### Preparation of Recombinant Proteins and Their Polyclonal Antibodies

The ORF of *Cg*cGAS, *Cg*IRF-8, and the CBD (c-di-GMP-binding domain) sequences of *Cg*STING was amplified using their respective primers (**Table S1**, P27–P32), and the PCR fragments were cloned into pET-30a, pMAL-c5x and pET-30a expression vector, respectively. The recombinant plasmids were transformed and expressed in *Escherichia coli* Transetta DE3. The recombinant proteins of *Cg*cGAS (r*Cg*cGAS) and CBD in *Cg*STING (r*Cg*STING-CBD) and *Cg*IFNLP (r*Cg*IFNLP) with two His tag and *Cg*IRF-8 (r*Cg*IRF-8) with MBP tag were purified by Ni^+^ or Maltose affinity chromatography and desalted by extensive dialysis.

The purified proteins of r*Cg*cGAS, r*Cg*IRF-8, and r*Cg*IFNLP were injected into six-weeks female mice to acquire polyclonal antibody as in previous description, respectively (33). Rabbit polyclonal antibody of *Cg*STING was obtained and purified by Gene Universal (34). The specificity of polyclonal antibodies was examined by Western blot. The hemocyte proteins were extracted from oysters after Poly (I:C) stimulation, separated by SDS-PAGE, and observed by a standard Western blot procedure as described previously (33).

### Immunocytochemical Assay

The hemocytes collected from oysters were resuspended in L15 cell culture media (extra addition of 20.2 g/L NaCl, 0.54 g/L KCl, 0.6 g/L CaCl, 3.9 g/L MgCl_2_, and 1 g/L MgSO_4_) and deposited on dishes pre-coated with poly-L-lysine, and then incubated at 37°C for 1 h to adhere to the glass slides. The hemocytes on the object slides were fixed with 4% paraformaldehyde, washed with PBS, and permeabilized with 1% Triton X-100 for 5 min. After three times of PBS wash, the hemocytes were blocked with 3% (w/v) fetal bovine serum albumin (BSA) at 37°C for 30 min and incubated with the antibody [anti-*Cg*IRF-8, anti-*Cg*cGAS, 1:500 (v/v) in 3% BSA] at 37°C for 1 h. Then the samples were washed with PBS and incubated with Alexa Fluer 488-labeled goat-anti-mouse antibody (Solarbio life sciences, China) diluted at 1:1,000 (v/v) with 3% BSA at 37°C in the dark for 1 h. The DAPI dihydrochloride (1 mg/ml in PBS; Solarbio Life Sciences, China) was added to stain the nucleus for 5 min followed by three times wash with PBS. Fluorescence was observed using Carl Zeiss Axio Imager A2 microscope (Carl Zeiss, Germany).

### PAMP Binding Assay

The PAMP binding activity of r*Cg*cGAS and r*Cg*STING-CBD was examined by enzyme-linked immunosorbent assay (35) as described previously (36). Briefly, the 96-well microliter plates were coated with dsEGFP (dsDNA) and Poly (I:C) at 4°C for 12 h, respectively. The plate was blocked with 3% BSA and incubated with 100 μl of r*Cg*cGAS or r*Cg*STING-CBD at 18°C for 3 h. Blank plasmid coded recombination protein rTrx was added in separated wells as a negative control at the same time. After three times of washing with TBST, anti-His tag antibody (ABclonal, USA) and diluted HRP Goat-anti-mouse Ig-alkaline phosphatase conjugate (ABclonal, USA) were added successively and incubated at 37°C for 1 h. After the final wash with TBST, 100 μl of TMB (dihydrochloride) (Solarbio, USA) was added and incubated at room temperature in dark for 15 min. The reaction was stopped by adding 1 M HCl (100 μl/well), and the absorbance at OD_450_ was measured by Infinite M1000 PRO (Tecan, Switzerland). The dissociation constant (Kd) was calculated using GraphPad Prism 5 with non-linear regression curve fit and a one-site binding model analysis.

### RNA Interference

The 3′-terminal cDNA sequences of *Cg*cGAS, *Cg*STING, *Cg*TBK1, *Cg*IFNLP, and EGFP were amplified by the primers Fi and Ri linked to the T7 promoter (**Table S1**, P33–42) as templates for the synthesis of dsRNA. The dsRNA was synthesized using T7 polymerase according to the instruction of manufacturer (Takara, China). The dsRNA (100 μg) was injected into adductor muscle of each oyster (nine oysters/group). To strengthen the effect of RNA interference, the second injection was carried out at 12 h after the first injection. After the second injection, the oysters were stimulated with Poly (I:C) (1 mg/ml, 0.1 ml). The hemocytes from three individuals were pooled together as one sample and centrifuged to harvest the hemocytes at 24 h after the second injection. The total RNA was extracted from the hemocytes, and the expression level of target genes was assessed by qRT-PCR.

### Statistical Analysis

All the data were expressed as mean ± SEM unless otherwise specified. Statistical analysis was conducted with an ANOVA followed by the Tukey *t*-test using GraphPad Prism 5.0 software and SPSS software.

## Results

### The Expression of ISGs After Poly (I:C) Stimulation and CgIFNLP RNAi

**Figure 1:**
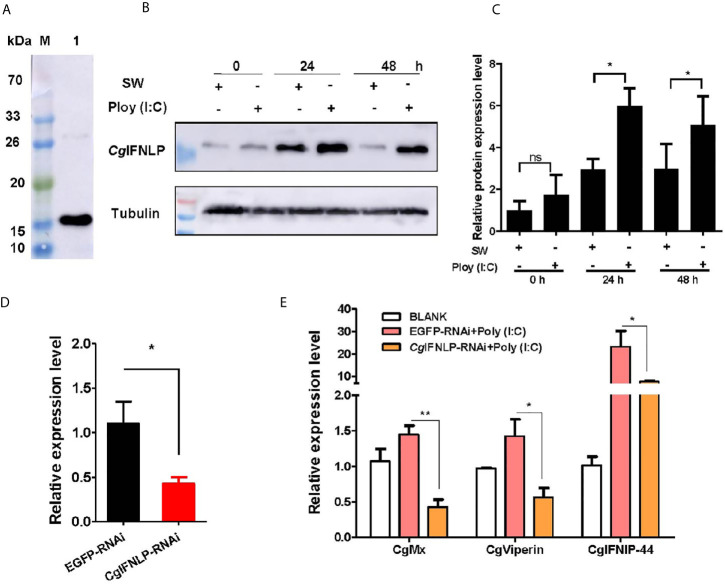
The expression of *Cg*ISGs in *Cg*IFNLP–RNAi oysters. **(A)** The specificity of polyclonal antibody for *Cg*IFNLP examined by Western blot. **(B)** The protein level of *Cg*IFNLP in hemocytes after Poly (I:C) stimulation. **(C)** The grayscale analysis of *Cg*IFNLP protein after Poly (I:C) stimulation using ImageJ software. **(D)** The efficiency of *Cg*IFNLP–RNAi in hemocytes (EGFP–RNAi was used as control). **(E)** The mRNA expressions of *Cg*Mx, *Cg*Viperin, and *Cg*IFNIP-44 in *Cg*IFNLP–RNAi oysters after Poly (I:C) stimulation. Vertical bars represent the mean ± SEM (N = 3). *p < 0.05; **p < 0.01; ns, not significant.

The specificity of polyclonal antibody against *Cg*IFNLP and the abundance of *Cg*IFNLP in hemolymph were examined by Western blot. A single band about 15 kDa with the high specificity was observed, which was consistent with the predicted molecular mass of *Cg*IFNLP (**Figure 1A**). The intensity of *Cg*IFNLP band in hemocyte proteins increased after Poly (I:C) stimulation (**Figures 1B, C** **)**. The dsRNA targeted *Cg*IFNLP was applied to suppress the *Cg*IFNLP expression, and its RNA expression level decreased to 0.39-fold of that in the EGFP-RNAi group (*p* < 0.05) (**Figure 1D**). The mRNA expression levels of *Cg*ISG-like molecules, including *Cg*Mx, *Cg*Viperin, and *Cg*IFNIP-44 (interferon-induced protein 44) in EGFP-RNAi group were up-regulated after Poly (I:C) stimulation, but they were suppressed in *Cg*IFNLP–RNAi oysters, which was 0.30-fold (*p* < 0.01), 0.40-fold (*p* < 0.05), and 0.33-fold (*p* < 0.05) of that in the EGFP-RNAi group, respectively (**Figure 1E**).

### CgcGAS and Its Activity to Recognize Viral Nucleic Acid

**Figure 2:**
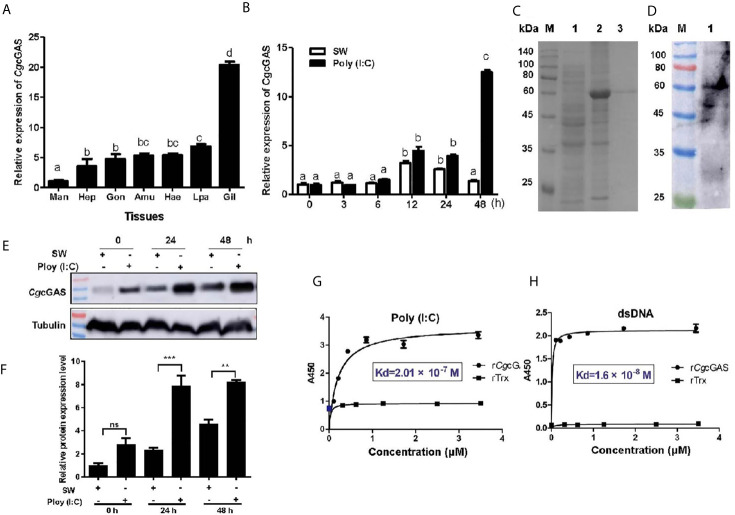
The binding activity of *Cg*cGAS to viral nucleic acid. **(A)** The tissue distribution of *Cg*cGAS mRNA. Man, mantle; Hep, hepatopancrease; Gon, gonad; Amu, adductor muscle; He, hemolymph; Lpa, labial palp; Gil, gill. **(B)** The temporal mRNA expression of the *Cg*cGAS after Poly (I:C) stimulation in hemocytes. **(C)** The recombination protein of *Cg*cGAS. Lane M: standard protein molecular weight marker; Lane 1: negative control (without induction); Lane 2: induced r*Cg*cGAS; Lane 3: purified r*Cg*cGAS. **(D)** Lane 1: the specificity of polyclonal antibody for *Cg*cGAS detected by Western blot. **(E)** The protein level of *Cg*cGAS after Poly (I:C) stimulation. **(F)** The statistical analysis of *Cg*cGAS protein level using ImageJ. **(G)** The binding activity of r*Cg*cGAS towards Poly (I:C) and **(H)** dsDNA. A450 was the absorbance value at 450 nm, and the Kd values were calculated by GraphPad Prism 5. **p < 0.01; ***p < 0.001; ns, not significant. The different letters show the existence significant differences comparing with other groups (*p* < 0.05).

A cGAS homolog (designated as *Cg*cGAS) was identified from *C. gigas*. The ORF of *Cg*cGAS was of 1,773 bp encoding a putative polypeptide of 590 amino acids (**Figure S1A**). There were a Mab-21 and three consecutive Zinc finger domains in the predicted *Cg*cGAS protein (**Figure S1A**). *Cg*cGAS shared the conserved key amino acids with cGAS sequences from *Mus musculu*, *Homo sapiens*, *Danio rerio*, and *Drosophila melanogaster* (**Figure S1B**). In the phylogenetic tree, *Cg*cGAS was firstly clustered with cGAS from *C. virginica* as a sister branch of the cGAS from *Drosophila* (**Figure S1C**). The mRNA transcripts of *Cg*cGAS were detected in all the tested tissues with the highest level in gill, which was nearly 20-fold higher than that in mantle (*p* < 0.05) (**Figure 2A**). The expression level of *Cg*cGAS mRNA in hemocytes increased significantly at 12 h with highest expression at 48 h (13.08-fold of that at 0 h, *p* < 0.05) after Poly (I:C) stimulation (**Figure 2B**). The recombinant protein of *Cg*cGAS (r*Cg*cGAS) was purified using the Ni-NATA affinity chromatography and examined by 15% SDS-PAGE. An evident band with a molecular weight about 58 kDa was observed, corresponding to *Cg*cGAS with His-tag (6 kDa) (lane 3 in **Figure 2C**). The purified r*Cg*cGAS was used to prepare polyclonal antibody, and a distinct band about 60 kDa was revealed in hemocyte protein by Western blot (**Figure 2D**). The abundance of *Cg*cGAS protein in hemocytes increased significantly at 24 and 48 h after Poly (I:C) stimulation (**Figures 2E, F**). The binding activity of r*Cg*cGAS toward dsRNA and dsDNA was examined by ELISA. r*Cg*cGAS could directly bind dsRNA and dsDNA in a concentration dependent manner with a saturable process from 0 to 3.5 μM (**Figures 2G, H** **)**. The apparent Kd values of r*Cg*cGAS toward dsRNA and dsDNA, calculated from the saturation curve, were 2.01 × 10^−7^ and 1.6 × 10^−8^, respectively (**Figures 2G, H** **)**.

### The Regulation of CgcGAS on CgIFNLP Activation and the Nuclear Translocation of CgIRF8

**Figure 3:**
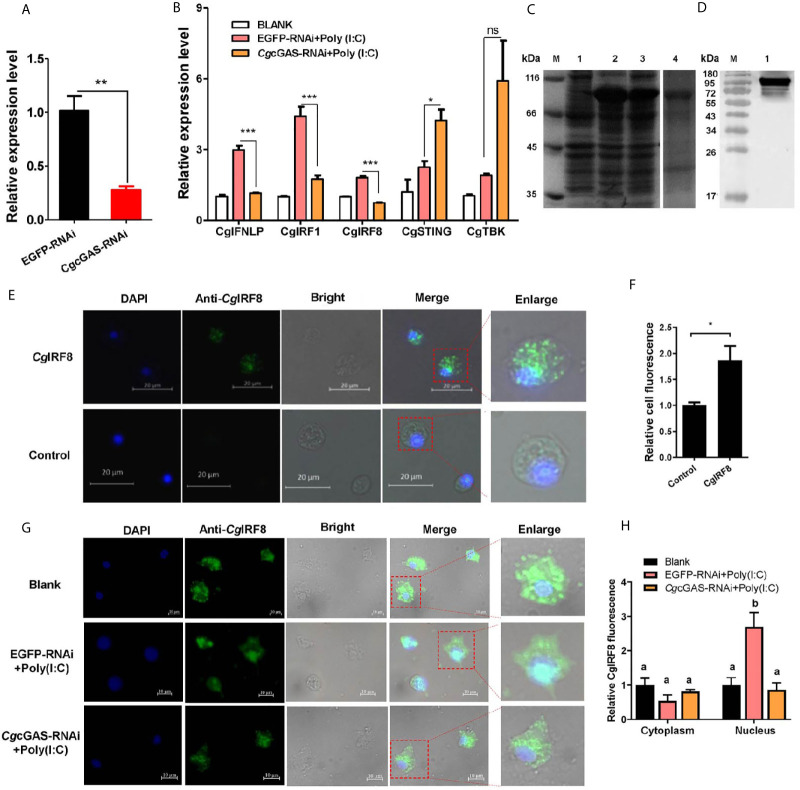
Regulation of *Cg*cGAS on the expression of *Cg*IFNLP and nuclear translocation of *Cg*IRF-8. **(A)** The efficiency of *Cg*cGAS–RNAi in hemocytes. EGFP–RNAi was used as control. **(B)** The mRNA expressions of *Cg*IFNLP, *Cg*IRF-1, *Cg*IRF-8, *Cg*STING, and *Cg*TBK1 in *Cg*cGAS–RNAi oysters after Poly (I:C) stimulation. **(C)** The recombination protein of *Cg*IRF-8. Lane M: standard protein molecular weight marker; Lane 1: negative control (without induction); Lane 2: induced r*Cg*IRF-8; Lane 3: Supernatant of lysate; Lane 4: purified r*Cg*cGAS. **(D)** Lane 1: specificity of polyclonal antibody for *Cg*IRF-8 examined by Western blot. **(E)** The subcellular localization of *Cg*IRF-8 (negative serum as control) in hemocytes and **(F)** the statistical analysis of relative fluorescence intensity of *Cg*IRF-8 using ImageJ. **(G)** The subcellular localization of *Cg*IRF-8 in *Cg*cGAS–RNAi hemocytes after Poly (I:C) stimulation and **(H)** the statistical analysis of fluorescence intensity using ImageJ. Nucleus staining with DAPI is shown in blue signal; anti-*Cg*IRF-8 conjugated to Alexa-fluor 488 is shown in green signal. Vertical bars represent the mean ± SEM. (N = 3). *p < 0.05; **p < 0.01; ***p < 0.001; ns, not significant. The different letters show the existence significant differences comparing with other groups (*p* < 0.05).

In order to identify the potential immune function of *Cg*cGAS in the regulation of *Cg*IFNLP, the dsRNA of *Cg*cGAS was employed to interfere *Cg*cGAS expression. The mRNA transcript of *Cg*cGAS was down-regulated significantly after dsRNA injection, which was 0.27-fold of that in the EGFP group (*p* < 0.01, **Figure 3A**). All the mRNA expression levels of *Cg*IFNLP, *Cg*IRF1, and *Cg*IRF8 decreased obviously in the *Cg*cGAS-RNAi group after Poly (I:C) treatment, which was 0.39-fold (*p* < 0.001), 0.40-fold (*p* < 0.001), and 0.41-fold (*p* < 0.001) of that in the EGFP-RNAi group, respectively (**Figure 3B**). Unexpectedly, the mRNA expression level of *Cg*STING and *Cg*TBK1 increased to some extent after *Cg*cGAS was knocked down, which was 1.88-fold (*p* < 0.05) and 3.1-fold (ns) of that in EGFP-RNAi oysters after Poly (I:C) treatment, respectively (**Figure 3B**). The recombinant proteins of *Cg*IRF8 (r*Cg*IRF8) were obtained by prokaryotic expression with a molecular weight about 97 kDa (containing MBP-tag of 42 kDa) (**Figure 3C**). The polyclonal antibody of r*Cg*IRF8 was further prepared, and its specificity was verified by Western blot (**Figure 3D**). The subcellular localization of *Cg*IRF8 in oyster hemocytes was determined by immunocytochemical assay. The positive signals of *Cg*IRF8 were observed in green, which were mainly distributed in the cytoplasm (**Figures 3E, F** **)**. After Poly (I:C) stimulation, the green signals of *Cg*IRF8 in hemocyte nucleus increased significantly compared to those in the blank group, which were overlapped with blue signals of nuclei stained by DAPI (**Figures 3G, H** **)**. However, the green signals of *Cg*IRF8 were still mainly distributed in the hemocyte cytoplasm of *Cg*cGAS-RNAi oysters after Poly (I:C) stimulation (**Figures 3G, H** **)**.

### CgSTING and Its Recognition to Viral Nucleic Acid

**Figure 4:**
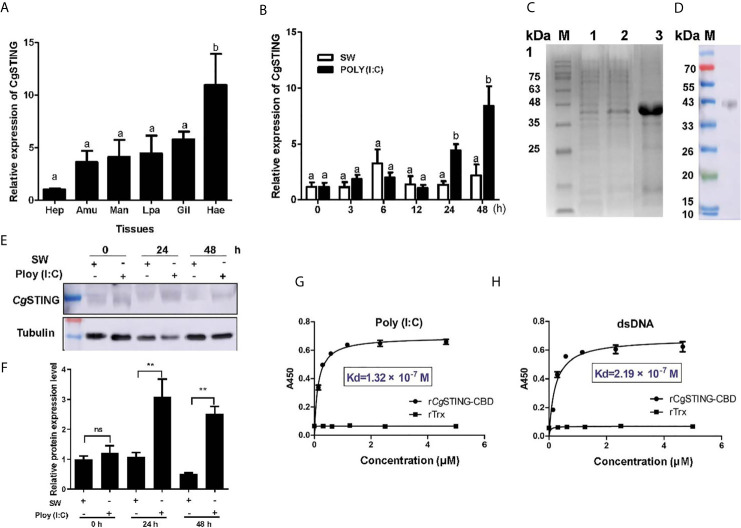
The binding activity of *Cg*STING to viral nucleic acid. **(A)** The tissues distribution of the *Cg*STING mRNA. Man, mantle; Hep, hepatopancrease; Amu, adductor muscle; He, hemolymph; Lpa, labial palp; Gil, gill. **(B)** The temporal expression profile of *Cg*STING mRNA after Poly (I:C) stimulation. **(C)** The recombination protein of *Cg*STING-CBD. Lane M: standard protein molecular weight marker; Lane 1: negative control (without induction); Lane 2: induced r*Cg*STING; Lane 3: purified r*Cg*STING. **(D)** Lane 1: Specificity detection for polyclonal antibody of *Cg*STING-CBD by Western blot. **(E)** The protein level of *Cg*STING after Poly (I:C) stimulation. **(F)** The statistical analysis of *Cg*STING protein level using ImageJ. **(G)** The binding activity of r*Cg*STING-CBD to Poly (I:C) and **(H)** dsDNA. A450 was the absorbance value at 450 nm, and the Kd values were calculated by GraphPad Prism 5. The different letters show the existence significant differences comparing with other groups (*p* < 0.05). **p < 0.01; ns, not significant.

A STING homolog (designated as *Cg*STING) was identified in *C. gigas* with an ORF of 1,191 bp encoding a peptide of 396 amino acids (**Figure S2A**). There was a Toll/IL-1R homologous and a C-di-GMP-bing domain (CBD) in the predicated *Cg*STING protein, which was consistent with the STINGs from other animals (**Figure S2B**). The mRNA transcripts of *Cg*STING were highly expressed in hemocytes, which were 10.80-fold (*p* < 0.01) of those in hepatopancrease (**Figure 4A**). No significant difference of expression level was observed in other tissues (**Figure 4A**). After Poly (I:C) stimulation, the mRNA expression of *Cg*STING in hemocytes increased significantly at 24 and 48 h, which was 3.81 and 7.25-fold (*p* < 0.001) of that at 0 h (**Figure 4B**), respectively. The recombinant protein of *Cg*STING-CBD with His-tag was obtained by prokaryotic expression and purified using the Ni-NATA affinity chromatography (**Figure 4C**). The purified r*Cg*STING-CBD was further used to prepare polyclonal antibody, whose specificity was verified with hemocyte protein by Western blot, and a single distinct band about 43 kDa was observed (**Figure 4D**). The *Cg*STING protein in hemocytes also increased after Poly (I:C) stimulation at 24 and 48 h (**Figures 4E, F** **)**. The PAMP binding assay showed that r*Cg*STING-CBD could directly bind dsRNA and dsDNA in a concentration dependent manner with a saturable process from 0 to 5 μM (**Figures 4G, H** **)**. The Kd values of r*Cg*STING-CBD toward dsRNA and dsDNA were 1.32 × 10^−7^ and 2.19 × 10^−7^, respectively (**Figures 4G, H** **)**.

### The Regulation of CgSTING on CgIFNLP Activation and Nuclear Translocation of CgIRF8

**Figure 5:**
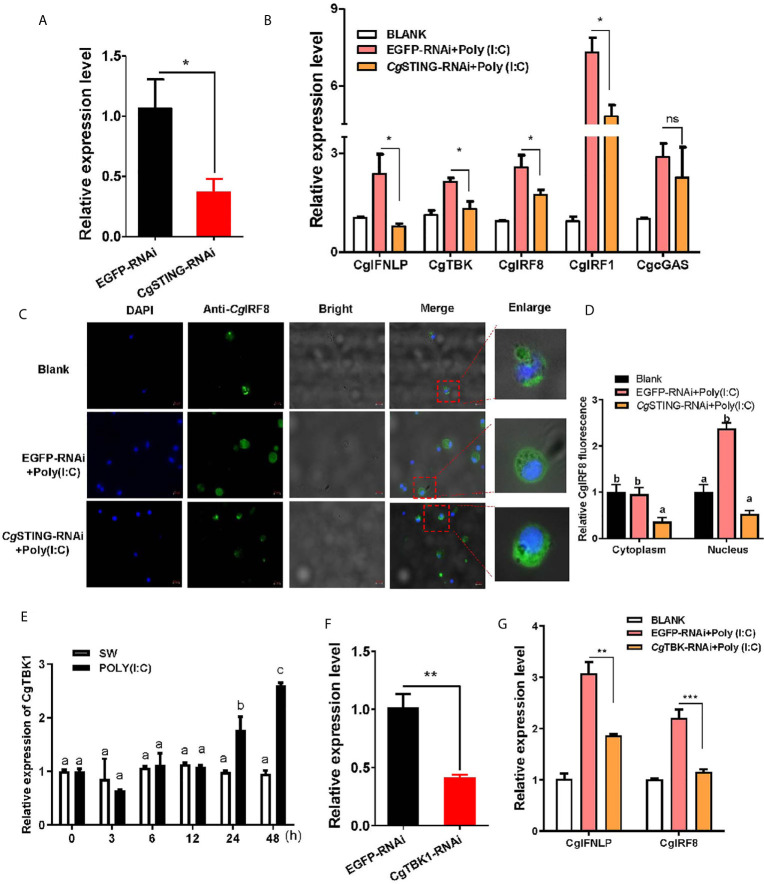
The regulation of *Cg*STING and *Cg*TBK1 on the expression of *Cg*IFNLP and IRFs. **(A)** The efficiency of *Cg*STING-RNAi in hemocytes (EGFP–RNAi was used as control). **(B)** The mRNA expressions of *Cg*IFNLP, *Cg*TBK1, *Cg*IRF-1, *Cg*IRF-8, and *Cg*cGAS in *Cg*STING–RNAi oysters after Poly (I:C) stimulation were detected by qRT-PCR. **(C)** The subcellular localization of *Cg*IRF-8 protein in hemocytes of *Cg*STING–RNAi oyster after Poly (I:C) stimulation. **(D)** The statistical analysis of relative fluorescence intensity of *Cg*IRF-8 using ImageJ. Nucleus staining with DAPI was shown in blue signal; anti-*Cg*IRF-8 conjugated to Alexa-fluor 488 was shown in green signal. **(E)** Temporal expression of *Cg*TBK1 mRNA after Poly (I:C) stimulation. **(F)** The efficiency of *Cg*TBK1–RNAi in hemocytes. **(G)** The mRNA expressions of *Cg*IFNLP and *Cg*IRF-8 in *Cg*TBK1–RNAi oysters after Poly (I:C) stimulation. Vertical bars represent the mean ± SEM (N = 3). *p < 0.05; **p < 0.01; ***p < 0.001; ns, not significant. The different letters show the existence significant differences comparing with other groups (*p* < 0.05).

The mRNA expression of *Cg*IFNLP, *Cg*TBK1, *Cg*IRF1, and *Cg*IRF8 was examined after *Cg*STING was knocked down by RNAi with *Cg*STING-dsRNA. The expression level of *Cg*STING mRNA in hemocytes decreased at 24 h after the injection with *Cg*STING-dsRNA, which was 0.34-fold of that in EGFP-RNAi oysters (**Figures 5A**). The mRNA expressions of *Cg*IFNLP, *Cg*TBK1, *Cg*IRF1, and *Cg*IRF8 in hemocytes were all suppressed in *Cg*STING-RNAi oysters after Poly (I:C) stimulation, which was 0.33-fold (*p* < 0.05), 0.62-fold (*p* < 0.05), 0.66-fold (*p* < 0.05), and 0.67-fold (*p* < 0.05) of those in EGFP-RNAi oysters, respectively (**Figure 5B**). No significant change of *Cg*cGAS expression was observed in *Cg*STING-RNAi oysters after Poly (I:C) stimulation (**Figure 5B**). The subcellular localization of *Cg*IRF8 in hemocytes of *Cg*STING-RNAi oysters was detected by immunocytochemical assay with anti-*Cg*IRF8 polyclonal antibody. The positive signals of *Cg*IRF8 labeled with Alexa Fluer 488 were observed in green, which were mainly distributed in the cytoplasm of oyster hemocytes in blank group. After Poly (I:C) stimulation, the green signals of *Cg*IRF8 were mainly located in hemocyte nucleus of EGFP-RNAi oysters. In *Cg*STING-RNAi oysters, the green signals were still mainly observed in hemocyte cytoplasm with few distributions in the nucleus (**Figures 5C, D** **)**. The expression of *Cg*TBK1 in hemocytes increased after Poly (I:C) stimulation at 24 and 48h (**Figure 5E**). After the injection with dsRNA of *Cg*TBK1, its mRNA expression level decreased to 0.41-fold of that in EGFP-RNAi group (*p* < 0.01) (**Figure 5F**). In the EGFP-RNAi group, the expression levels of *Cg*IRF8 and *Cg*IFNLP mRNA in hemocytes both increased after Poly (I:C) stimulation. In *Cg*TBK1–RNAi group, the increase of *Cg*IRF8 and *Cg*IFNLP expressions induced by Poly (I:C) injection was inhibited, which was 0.52-fold (*p* < 0.001) and 0.61-fold (*p* < 0.01) of that in the EGFP-RNAi group, respectively (**Figure 5G**).

## Discussion

IFNs are proteins or glycoproteins that spontaneously orchestrate complex cell-intrinsic and cell-extrinsic antiviral defenses through their effects on the intracellular events of viral cycle. Since IFNs were first defined in 1957, a large number of studies have been carried out on their molecular structural features, expression patterns, signaling pathways, and myriad roles in innate and adaptive immunity. So far, IFNs have been widely discovered in almost all kinds of vertebrates from fish to mammals (16, 37). Therefore, IFNs with conserved sequence are deemed to evolutionarily derive from teleosts (17). In addition to the classic antiviral function, the recombinant fish IFN*φ*1 were identified to possess broad-spectrum direct antibacterial activities, indicating IFNs’ multiple functions and importance in host defense (38). Recently, there have been increasing pieces of evidence on IFN-like molecule mediated antiviral mechanism in invertebrate (20). In arthropod, a novel viral infection-inducible peptide, Vago, has been characterized in *Drosophila*, *Culex*, and *L. vannamei* (21, 22, 24). Although these Vagos share low sequence similarity with IFNs from vertebrates, they are identified with the similar functions as Type I IFNs in vertebrates. They activate the semblable JAK–STAT pathway to block viral reproduction and protect arthropod from virus infection (21). Even an IFN-like protein and a cytokine receptor-like 3 have been identified from mollusk *C. gigas* (30, 31), the existence of IFN-like system and the detailed molecular mechanism underlying IFN-like mediated antiviral responses remains bewildered in primitive invertebrates. In this study, the signal pathways to mediate the expression of *Cg*IFNLP were investigated to comprehensively understand its involvement in antiviral immune responses in *C. gigas.*

In response to viral infections, vertebrate IFNs up-regulate the expression of hundreds of ISGs, whose cumulative action can potently inhibit the replication of virus (39). In the present study, the protein level of *Cg*IFNLP in hemocytes was observed to increase after Poly (I:C) stimulation. In order to confirm the activation mechanism of IFN signaling pathways in oysters, RNAi was applied to inhibit the expression of *Cg*IFNLP. The expression of *Cg*ISGs including *Cg*Mx, *Cg*Viperin, and *Cg*IFNIP-44 was suppressed after *Cg*IFNLP was knocked down. As a classic ISG, MX1 acts prior to genome replication at an early post entry step of the virus life cycle, while viperin inhibits both viral egress and the replication of multiple viruses, and IFNIP-44 has been shown to restrict infection involving the reduction of viral genome transcription or replication (39–41). The results of *Cg*IFNLP–RNAi indicated that *Cg*IFNLP displayed similar characteristics with vertebrate IFNs to resist viral infection dependent on ISG release (2).

In vertebrates, virus-derived DNA is sensed by cGAS in the cytosol, which synthesizes the second messenger 2′3′-cGAMP to bind and activate the signaling adaptor STING (42). The activated STING initiates a downstream signaling event by recruiting and activating TBK1 or IKK, which leads to the phosphorylation of IRF3 to initiate the expression of IFNs and I*κ*B family of inhibitors of transcription factor NF-*κ*B, respectively (13). The cGAS homologs have been reported to be present in lots of vertebrate species and show similar structures with human or murine cGAS (11). In the present study, both cGAS and STING homologs were identified in *C. gigas*, and they shared sequence conservation with their homologs from vertebrates. The origins of cGAS and STING were considered to be traced back to a choanoflagellate *Monosiga brevicollis*, the closest relative of metazoans (43). However, it was found that the cGAS ortholog in *Drosophila* did not play a role during *Listeria* or IIV6 infection, which was speculated to be ascribed to the lack of the zinc-ribbon domain and a positively charged N terminus functionally important for DNA binding (44). Interestingly, a functional cGAS–STING pathway has been reported in bacteria *Vibrio cholerae*, which suggested that the eukaryotic cGAS–STING antiviral pathway has ancient evolutionary roots stemming from microbial defenses against phages (45). The cGAS–STING pathway was also confirmed to exist in the sea anemone *Nematostella vectensis* (46). Unlike the cGAS in mammal and arthropod, *Cg*cGAS in *C. gigas* was able to bind both dsRNA and dsDNA with higher combining capacity with dsRNA and sensitivity with dsDNA. However, the cGAS homologs in invertebrates have been speculated to lose the function as DNA sensors, and act a nucleotidyl transferase to produce cGAMP and other cyclic dinucleotides (CDNs) (43). The recognition capability of *Cg*cGAS with double-stranded nucleic acid might benefit from its conserved Zinc finger domains and critical amino acid residues. The expression of *Cg*cGAS was highly induced after Poly (I:C) stimulation, indicating its involvement in antiviral immunity of *C. gigas.* After *Cg*cGAS was knocked down by RNAi, the expression of *Cg*IFNLP, *Cg*IRF-1 and -8 in hemocytes was reduced, which demonstrated the important roles of *Cg*cGAS in *Cg*IFNLP mediating antiviral immune response. Consistently, induction of antiviral IFNs is the major outcome of cGAS–STING activation in vertebrates (42). Moreover, the inhibition of *Cg*cGAS expression suppressed the nuclear translocation of *Cg*IRF-8 after Poly (I:C) stimulation, revealing that the *Cg*cGAS might induce the *Cg*IFNLP expression through mediating the nuclear translocation of *Cg*IRF-8. IRFs are the key transcriptional factors of IFN signaling, and a lot of IRFs have been discovered in invertebrates, such as *Branchiostoma belcheri tsingtauense* (47), *Litopenaeus vannamei* (23), *Pinctada fucata* (27), and *C. gigas* (48). Three IRFs (CgIRF-1a, -1b and -8) have been identified in *C. gigas* (48). *Cg*IRF-1a and *Cg*IRF-1b with typical IRF domain (also known as DNA-binding domain) have been certified to regulate the expression of *Cg*IFNLP as a transcriptional regulatory factor (26). In the present study, *Cg*IRF-8 was identified from *C. gigas* as a crucial factor for *Cg*cGAS, and its mRNA expression was induced after Poly (I:C) stimulation, indicating that both *Cg*IRF-1 and *Cg*IRF-8 are necessary for the regulation of *Cg*IFNLP expression. Unexpectedly, the expression of *Cg*STING and *Cg*TBK1 did not change significantly after *Cg*cGAS was knocked down, but increased after Poly (I:C) stimulation in *Cg*cGAS-RNAi oysters, which suggested that *Cg*STING and *Cg*TBK1 were not the downstream molecules of *Cg*cGAS in *C. gigas*.

Recently, the functional STING homologs were identified in bacteria. They were functional cyclic dinucleotide receptors and showed TIR–STING fusion structure, which confirmed the STING cyclic dinucleotide sensing originated in bacteria (49). Together with the cGAS homologs in prokaryotic antiviral immunity, the functional cGAS–STING signaling might arise from an ancient mechanism of defense against bacteriophages. Moreover, the crystal structure of *C. gigas* STINGs was also analyzed, which showed conserved TIR–STING fusions and cyclic dinucleotide binding activity (49). Consistently, in the present study, the *Cg*STING from *C. gigas* displayed a similar TIR–STING fusion structure shown as TIR and CBD domains. The CBD of *Cg*STING (r*Cg*STING-CBD) could bind the Poly (I:C) and dsDNA *in vitro*. In mammals, the central CBD of STING was determined to mediate the binding to CDNs from cGAS (50). However, the relatively conservative domain in *Cg*STING suggested that it could act as a nucleic acid identifier, implying the largely elusive and diversiform functions of STINGs from invertebrates. Furthermore, the mRNA expression of *Cg*STING was found to increase after Poly (I:C) stimulation, indicating its involvement in oyster antiviral immunity. Increasing evidence suggested that the ancestral functions of STING might be related to the activation of antibacterial immunity (13), such as the STING in *Litopenaeus vannamei* (*Lv*STING) and *Drosophila* (*dm*STING), which were reported to be involved in the innate immune response against bacterial infection (44, 51). In the present study, after *Cg*STING was knocked down by RNAi, the expression level of *Cg*IFNLP, *Cg*TBK1, *Cg*IRF-1, and *Cg*IRF-8 mRNA, as well as the nuclear translocation *Cg*IRF-8 induced by Poly (I:C) was significantly suppressed. These results suggested that the *Cg*STING also mediated the expression of *Cg*IFNLP and *Cg*IRFs, which was consistent with *Cg*cGAS. However, different from the observation in mammals, the *Cg*STING and *Cg*TBK1 were not the downstream molecules of *Cg*cGAS. The homologs of STING in invertebrate are postulated to loss the function to induce innate immune response against infection for the lack of a carboxy-terminal tail (CTT) domain which is the essential domain for mammalian STING to recruit the critical downstream TBK1 and IRF3 signaling components (43, 50). However, *Cg*TBK1 from *C. gigas* was reported to interact with *Cg*STING in HEK293T cells, providing the evidence that *Cg*TBK1 could be activated by direct binding to *Cg*STING (52). Furthermore, the expression of *Cg*IFNLP and *Cg*IRF-8 in *Cg*TBK1–RNAi oysters also decreased after Poly (I:C) stimulation. The results collectively illustrated that *Cg*STING might activate *Cg*IFNLP to cope with viral nucleic acid by binding *Cg*TBK1 and further inducing the expression and nuclear migration of *Cg*IRFs. Therefore, it was proposed that the *Cg*cGAS and *Cg*STING recognized the viral nucleic acid simultaneously and further activated the *Cg*IFNLP expression synergistically.

In summary, *Cg*cGAS and *Cg*STING were identified to recognize and bind dsRNA and dsDNA. They were involved in the response against Poly (I:C) stimulation and synergistically facilitated the *Cg*IRF-mediated *Cg*IFNLP production, which further induced the expression of *Cg*ISGs (**Figure S3**). The results defined a *Cg*cGAS/STING–TBK1–IRFs regulatory axis to mediate the primitive IFN-like antiviral mechanism in *C. gigas*.

## Data Availability Statement

The raw data supporting the conclusions of this article will be made available by the authors, without undue reservation.

## Author Contributions

XQ, YZ, and ZW designed, performed, and analyzed the experiments, participated in the design of the study, and drafted the manuscript. ZL and YL participated in the design of the study and discussed the results. LW and LS conceived of the study, coordinated the experiment, and helped draft the manuscript. All authors contributed to the article and approved the submitted version.

## Funding

This research was supported by National Key R&D Program (2018YFD0900502), grants (Nos. U1706204, 41961124009, 32002418) from National Science Foundation of China, earmarked fund (CARS-49) from Modern Agro-industry Technology Research System, the Fund for Outstanding Talents and Innovative Team of Agricultural Scientific Research, Key R&D Program of Liaoning Province (2017203004, 2017203001), Liaoning Climbing Scholar, the Distinguished Professor of Liaoning (XLYC1902012).

## Conflict of Interest

The authors declare that the research was conducted in the absence of any commercial or financial relationships that could be construed as a potential conflict of interest.

## Abbreviations

IFN, interferon; ISGs, IFN-stimulated genes; cGAS, cyclic GMP-AMP synthase; STING, stimulator of interferon gene; TBK1, TANK binding kinase1; IRF, interferon regulatory factors; Mx, myxovirus resistance; PAMPs, pathogen-associated molecular patterns; PRRs, pattern recognition receptors; TLRs, Toll like receptors; RIG-I, retinoic acid-inducible gene I; MDA5, melanoma differentiation associated protein 5; IKK*Ɛ*, Ikappa B Kinase *Ɛ*; IFNR-3, IFN receptor-like 3; CBD, c-di-GMP-binding domain.

## References

[1] Decker T Stockinger S Karaghiosoff M Muller M Kovarik P. Ifns and STATs in Innate Immunity to Microorganisms. J Clin Invest (2002) 109:1271–7. 10.1172/JCI0215770 PMC150987 12021240

[2] Villarino AV Kanno Y O’Shea JJ. Mechanisms and Consequences of Jak-STAT Signaling in the Immune System. Nat Immunol (2017) 18:374–84. 10.1038/ni.3691 PMC11565648 28323260

[3] Kotenko SV Gallagher G Baurin VV Lewis-Antes A Shen M Shah NK. IFN-Lambdas Mediate Antiviral Protection Through a Distinct Class II Cytokine Receptor Complex. Nat Immunol (2003) 4:69–77. 10.1038/ni875 12483210

[4] Gray PW Goeddel DV. Structure of the Human Immune Interferon Gene. Nature (1982) 298:859–63. 10.1038/298859a0 6180322

[5] Leu SW Shi L Xu C Zhao Y Liu B Li B. TLR4 Through IFN-β Promotes Low Molecular Mass Hyaluronan-Induced Neutrophil Apoptosis. J Immunol (Baltimore Md. 1950) (2011) 186:556–62. 10.4049/jimmunol.1001630 21098223

[6] Elion DL Cook RS. Harnessing RIG-I and Intrinsic Immunity in the Tumor Microenvironment for Therapeutic Cancer Treatment. Oncotarget (2018) 9:29007–17. 10.18632/oncotarget.25626 PMC6034747 29989043

[7] Iurescia S Fioretti D Rinaldi M. Targeting Cytosolic Nucleic Acid-Sensing Pathways for Cancer Immunotherapies. Front Immunol (2018) 9:711. 10.3389/fimmu.2018.00711 29686682 PMC5900005

[8] Tan X Sun L Chen J Chen ZJ. Detection of Microbial Infections Through Innate Immune Sensing of Nucleic Acids. Annu Rev Microbiol (2018) 72:447–78. 10.1146/annurev-micro-102215-095605 30200854

[9] Vanpouille-Box C Demaria S Formenti SC Galluzzi L. Cytosolic DNA Sensing in Organismal Tumor Control. Cancer Cell (2018) 34:361–78. 10.1016/j.ccell.2018.05.013 30216189

[10] Wu J Chen ZJ. Innate Immune Sensing and Signaling of Cytosolic Nucleic Acids. Annu Rev Immunol (2014) 32:461–88. 10.1146/annurev-immunol-032713-120156 24655297

[11] Sun L Wu J Du F Chen X Chen ZJ. Cyclic GMP-AMP Synthase is a Cytosolic DNA Sensor That Activates the Type I Interferon Pathway. Sci (New York N.Y.) (2013) 339:786–91. 10.1126/science.1232458 PMC3863629 23258413

[12] Ishikawa H Ma Z Barber GN. STING Regulates Intracellular DNA-mediated, Type I Interferon-Dependent Innate Immunity. Nature (2009) 461:788–92. 10.1038/nature08476 PMC4664154 19776740

[13] Margolis SR Wilson SC Vance RE. Evolutionary Origins of cGAS-STING Signaling. Trends Immunol (2017) 38:733–43. 10.1016/j.it.2017.03.004 28416447

[14] Mancino A Natoli G. Specificity and Function of IRF Family Transcription Factors: Insights From Genomics. J Interferon Cytokine Res Off J Int Soc Interferon Cytokine Res (2016) 36:462–9. 10.1089/jir.2016.0004 27379868

[15] Negishi H Taniguchi T Yanai H. The Interferon (Ifn) Class of Cytokines and the IFN Regulatory Factor (Irf) Transcription Factor Family. Cold Spring Harbor Perspect Biol (2018) 10:a028423. 10.1101/cshperspect.a028423 PMC6211389 28963109

[16] Langevin C Aleksejeva E Passoni G Palha N Levraud JP Boudinot P. The Antiviral Innate Immune Response in Fish: Evolution and Conservation of the IFN System. J Mol Biol (2013) 425:4904–20. 10.1016/j.jmb.2013.09.033 24075867

[17] Vilcek J. Fifty Years of Interferon Research: Aiming at a Moving Target. Immunity (2006) 25:343–8. 10.1016/j.immuni.2006.08.008 16979566

[18] Nakatani Y Takeda H Kohara Y Morishita S. Reconstruction of the Vertebrate Ancestral Genome Reveals Dynamic Genome Reorganization in Early Vertebrates. Genome Res (2007) 17:1254–65. 10.1101/gr.6316407 PMC1950894 17652425

[19] Secombes C Zou J. Evolution of Interferons and Interferon Receptors. Front Immunol (2017) 8:209. 10.3389/fimmu.2017.00209 28303139 PMC5332411

[20] Qiao X Wang L Song L. The Primitive Interferon-Like System and its Antiviral Function in Molluscs. Dev Comp Immunol (2021) 118:103997. 10.1016/j.dci.2021.103997 33444647

[21] Deddouche S Matt N Budd A Mueller S Kemp C Galiana-Arnoux D. The DExD/H-box Helicase Dicer-2 Mediates the Induction of Antiviral Activity in Drosophila. Nat Immunol (2008) 9:1425–32. 10.1038/ni.1664 18953338

[22] Paradkar PN Trinidad L Voysey R Duchemin JB Walker PJ. Secreted Vago Restricts West Nile Virus Infection in Culex Mosquito Cells by Activating the Jak-STAT Pathway. Proc Natl Acad Sci USA (2012) 109:18915–20. 10.1073/pnas.1205231109 PMC3503207 23027947

[23] Li C Li H Chen Y Chen Y Wang S Weng SP. Activation of Vago by Interferon Regulatory Factor (IRF) Suggests an Interferon System-Like Antiviral Mechanism in Shrimp. Sci Rep (2015) 5:15078. 10.1038/srep15078 26459861 PMC4602278

[24] De Zoysa M Kang HS Song YB Jee Y Lee YD Lee J. First Report of Invertebrate Mx: Cloning, Characterization and Expression Analysis of Mx cDNA in Disk Abalone (Haliotis Discus Discus). Fish Shellfish Immunol (2007) 23:86–96. 10.1016/j.fsi.2006.09.007 17097889

[25] Green TJ Speck P Geng L Raftos D Beard MR Helbig KJ. Oyster Viperin Retains Direct Antiviral Activity and its Transcription Occurs Via a Signalling Pathway Involving a Heat-Stable Haemolymph Protein. J Gen Virol (2015) 96:3587–97. 10.1099/jgv.0.000300 26407968

[26] Lu M Yang C Li M Yi Q Lu G Wu Y. A Conserved Interferon Regulation Factor 1 (IRF-1) From Pacific Oyster Crassostrea Gigas Functioned as an Activator of IFN Pathway. Fish Shellfish Immunol (2018) 76:68–77. 10.1016/j.fsi.2018.02.024 29458094

[27] Huang XD Liu WG Wang Q Zhao M Wu SZ Guan YY. Molecular Characterization of Interferon Regulatory Factor 2 (IRF-2) Homolog in Pearl Oyster Pinctada Fucata. Fish Shellfish Immunol (2013) 34:1279–86. 10.1016/j.fsi.2013.02.003 23422814

[28] Le Deuff RM Renault T. Purification and Partial Genome Characterization of a Herpes-Like Virus Infecting the Japanese Oyster, Crassostrea Gigas. J Gen Virol (1999) 80(Pt 5):1317–22. 10.1099/0022-1317-80-5-1317 10355779

[29] de Lorgeril J Lucasson A Petton B Toulza E Montagnani C Clerissi C. Immune-Suppression by OsHV-1 Viral Infection Causes Fatal Bacteraemia in Pacific Oysters. Nat Commun (2018) 9:4215. 10.1038/s41467-018-06659-3 30310074 PMC6182001

[30] Zhang R Liu R Wang W Xin L Wang L Li C. Identification and Functional Analysis of a Novel IFN-Like Protein (CgIFNLP) in Crassostrea Gigas. Fish Shellfish Immunol (2015) 44:547–54. 10.1016/j.fsi.2015.03.015 25812419

[31] Zhang R Liu R Xin L Chen H Li C Wang L. A CgIFNLP Receptor From Crassostrea Gigas and its Activation of the Related Genes in Human JAK/STAT Signaling Pathway. Dev Comp Immunol (2016) 65:98–106. 10.1016/j.dci.2016.06.010 27373517

[32] Wu Z Sun J Wang L Zong Y Han Z Yang W. CgSOCS6 Negatively Regulates the Expression of CgIL17s and CgDefh1 in the Pacific Oyster Crassostrea Gigas. Fish Shellfish Immunol (2019) 93:1084–92. 10.1016/j.fsi.2019.08.055 31449980

[33] Zong Y Liu Z Wu Z Han Z Wang L Song L. A Novel Globular C1q Domain Containing Protein (C1qDC-7) From Crassostrea Gigas Acts as Pattern Recognition Receptor With Broad Recognition Spectrum. Fish Shellfish Immunol (2019) 84:920–6. 10.1016/j.fsi.2018.10.079 30385248

[34] Gushchina LV Kwiatkowski TA Bhattacharya S Weisleder NL. Conserved Structural and Functional Aspects of the Tripartite Motif Gene Family Point Towards Therapeutic Applications in Multiple Diseases. Pharmacol Ther (2018) 185:12–25. 10.1016/j.pharmthera.2017.10.020 29097306 PMC5721676

[35] Wynne C Lazzari E Smith S McCarthy EM Gabhann JN Kallal LE. TRIM68 Negatively Regulates IFN-β Production by Degrading TRK Fused Gene, a Novel Driver of IFN-β Downstream of Anti-Viral Detection Systems. PLoS ONE (2014) 9:e101503. 10.1371/journal.pone.0101503 24999993 PMC4084880

[36] Liu Y Zhang P Wang W Dong M Wang M Gong C. A DM9-containing Protein From Oyster Crassostrea Gigas (CgDM9CP-2) Serves as a Multipotent Pattern Recognition Receptor. Dev Comp Immunol (2018) 84:315–26. 10.1016/j.dci.2018.03.003 29518405

[37] Gan Z Chen SN Huang B Hou J Nie P. Intronless and Intron-Containing Type I IFN Genes Coexist in Amphibian Xenopus Tropicalis: Insights Into the Origin and Evolution of Type I Ifns in Vertebrates. Dev Comp Immunol (2017) 67:166–76. 10.1016/j.dci.2016.10.007 27780747

[38] Xiao X Zhu W Zhang Y Liao Z Wu C Yang C. Broad-Spectrum Robust Direct Bactericidal Activity of Fish Ifnφ1 Reveals an Antimicrobial Peptide-Like Function for Type I Ifns in Vertebrates. J Immunol (2021) 206:1337–47. 10.4049/jimmunol.2000680 33568398

[39] Feng J Wickenhagen A Turnbull M Rezelj V Kreher F Tilston-Lunel N. Interferon-Stimulated Gene (Isg)-Expression Screening Reveals the Specific Antibunyaviral Activity of ISG20. J Virol (2018) 92:02140–17. 10.1128/JVI.02140-17 PMC6002717 29695422

[40] Helbig KJ Beard MR. The Role of Viperin in the Innate Antiviral Response. J Mol Biol (2014) 426:1210–9. 10.1016/j.jmb.2013.10.019 24157441

[41] Busse D Habgood-Coote D Clare S Brandt C Bassano I Kaforou M. Interferon-Induced Protein 44 and Interferon-Induced Protein 44-Like Restrict Replication of Respiratory Syncytial Virus. J Virol (2020) 94:00297–20. 10.1128/JVI.00297-20 PMC7459546 32611756

[42] Gamdzyk M Doycheva DM Araujo C Ocak U Luo Y Tang J. Cgas/STING Pathway Activation Contributes to Delayed Neurodegeneration in Neonatal Hypoxia-Ischemia Rat Model: Possible Involvement of LINE-1. Mol Neurobiol (2020) 57:2600–19. 10.1007/s12035-020-01904-7 PMC7260114 32253733

[43] Wu X Wu FH Wang X Wang L Siedow JN Zhang W. Molecular Evolutionary and Structural Analysis of the Cytosolic DNA Sensor cGAS and STING. Nucleic Acids Res (2014) 42:8243–57. 10.1093/nar/gku569 PMC4117786 24981511

[44] Martin M Hiroyasu A Guzman RM Roberts SA Goodman AG. Analysis of Drosophila Sting Reveals an Evolutionarily Conserved Antimicrobial Function. Cell Rep (2018) 23:3537–3550 e3536. 10.1016/j.celrep.2018.05.029 29924997 PMC6114933

[45] Cohen D Melamed S Millman A Shulman G Oppenheimer-Shaanan Y Kacen A. Cyclic GMP-AMP Signalling Protects Bacteria Against Viral Infection. Nature (2019) 574:691–5. 10.1038/s41586-019-1605-5 31533127

[46] Kranzusch PJ Wilson SC Lee AS Berger JM Doudna JA Vance RE. Ancient Origin of Cgas-STING Reveals Mechanism of Universal 2’,3’ cGAMP Signaling. Mol Cell (2015) 59:891–903. 10.1016/j.molcel.2015.07.022 26300263 PMC4575873

[47] Yuan S Zheng T Li P Yang R Ruan J Huang S. Characterization of Amphioxus Ifn Regulatory Factor Family Reveals an Archaic Signaling Framework for Innate Immune Response. J Immunol (2015) 195:5657–66. 10.4049/jimmunol.1501927 26573836

[48] Mao F Lin Y Zhou Y He Z Li J Zhang Y. Structural and Functional Analysis of Interferon Regulatory Factors (Irfs) Reveals a Novel Regulatory Model in an Invertebrate, Crassostrea Gigas. Dev Comp Immunol (2018) 89:14–22. 10.1016/j.dci.2018.07.027 30077552

[49] Morehouse B Govande A Millman A Keszei A Lowey B Ofir G. STING Cyclic Dinucleotide Sensing Originated in Bacteria. Nature (2020) 586:429–33. 10.1038/s41586-020-2719-5 PMC7572726 32877915

[50] Tanaka Y Chen ZJ. STING Specifies IRF3 Phosphorylation by TBK1 in the Cytosolic DNA Signaling Pathway. Sci Signaling (2012) 5:ra20. 10.1126/scisignal.2002521 PMC3549669 22394562

[51] Li H Wang S Lu K Yin B Xiao B Li S. An Invertebrate STING From Shrimp Activates an Innate Immune Defense Against Bacterial Infection. FEBS Lett (2017) 591:1010–7. 10.1002/1873-3468.12607 28236646

[52] Tang X Huang B Zhang L Li L Zhang G. TANK-Binding Kinase-1 Broadly Affects Oyster Immune Response to Bacteria and Viruses. Fish Shellfish Immunol (2016) 56:330–5. 10.1016/j.fsi.2016.07.011 27422757

