# Supplementary material for: The cGAS/STING–TBK1–IRF Regulatory Axis Orchestrates a Primitive Interferon-Like Antiviral Mechanism in Oyster
Source: Front Immunol. 2021 Jun 8;12:689783. doi: 10.3389/fimmu.2021.689783 (PMC8218723; doi:10.3389/fimmu.2021.689783)
Supplement: Supplementary file 1 [file DataSheet_1.doc]

***Supplementary Material***

**Contents：**

**Supplementary Figures 1-3**

**Supplementary Table 1**

**
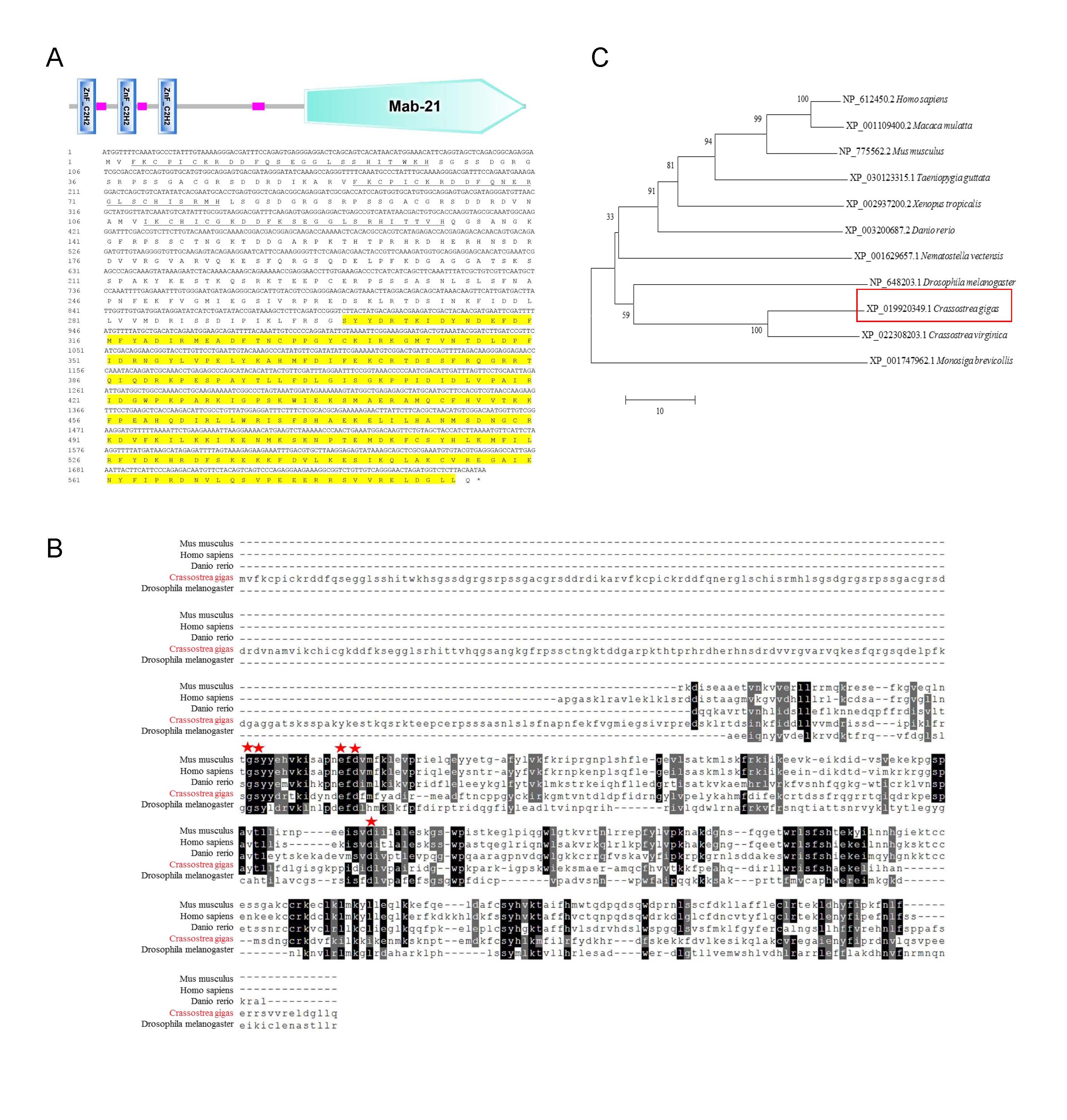
**

**Supplementary Figure 1. Sequence characters of *Cg*cGAS. (A)** Complete nucleotide sequence and the deduced structural domains of *Cg*cGAS proteins. The Zinc finger domains and Mab-21 are shown with black underlined and yellow highlight respectively. *(B)* Multiple sequence alignment of *Cg*cGAS with other cGAS members from *Mus musculu*, *Homo sapiens*, *Danio rerio*, *Drosophila melanogaster*. The conserved key amino acids are labeled with *. *(C)* Phylogenetic analysis of *Cg*cGAS with other cGAS from different animals.

**
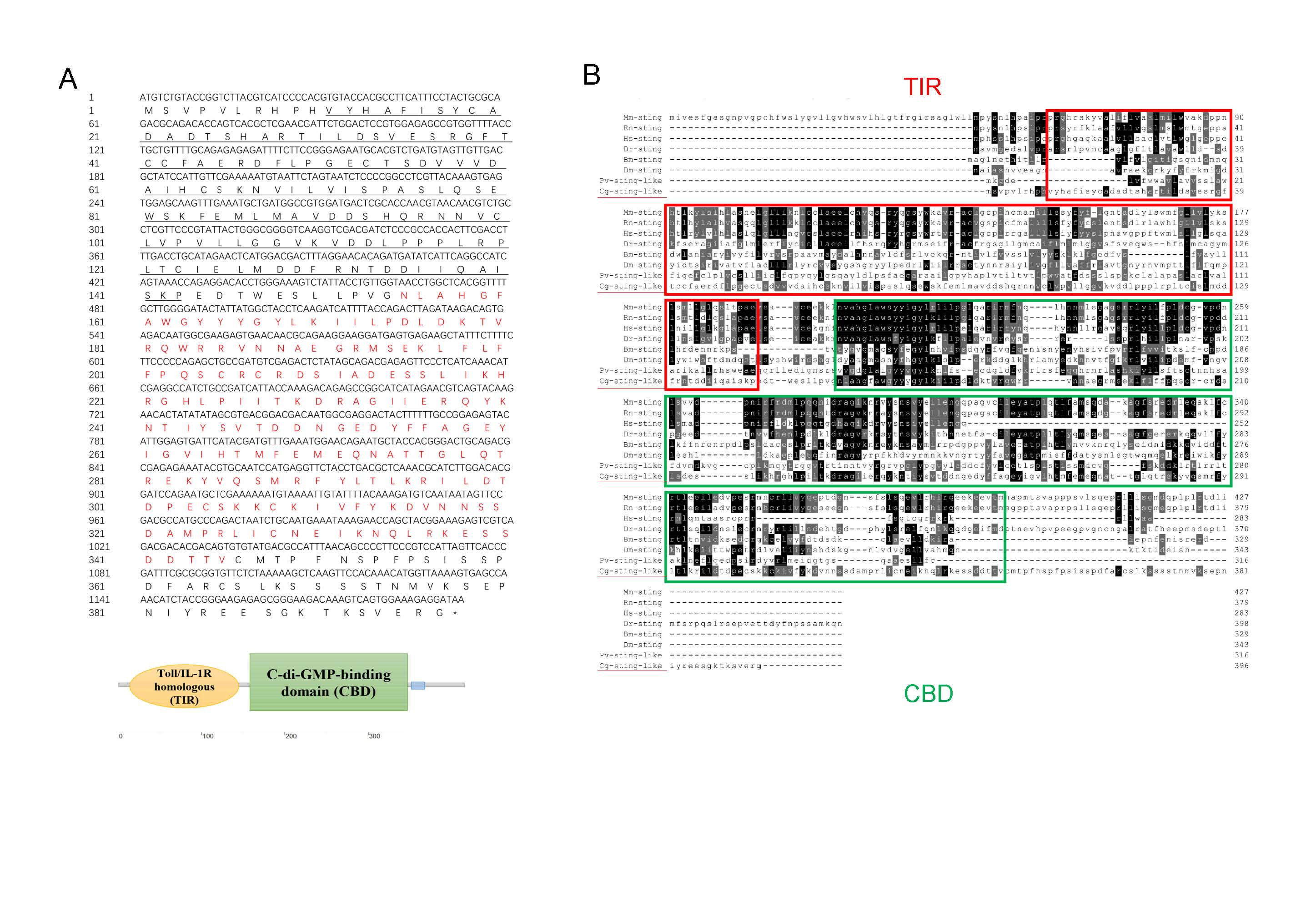
**

**Supplementary Figure 2. Sequence characters of *Cg*STING. (A)** Complete nucleotide sequence and the deduced structural domains of *Cg*STING. TIR and CBD are labeled by underline and red color, respectively. **(B)** Multiple sequence alignment of *Cg*STING with STINGs from other species, including *Mus musculus*, *Rattus norvegicus*, *Homo sapiens*, *Danio rerio*, *Drosophila melanogaster*, *Bombyx mori*, *Penaeus vannamei.* The conserved domains are labeled by box.


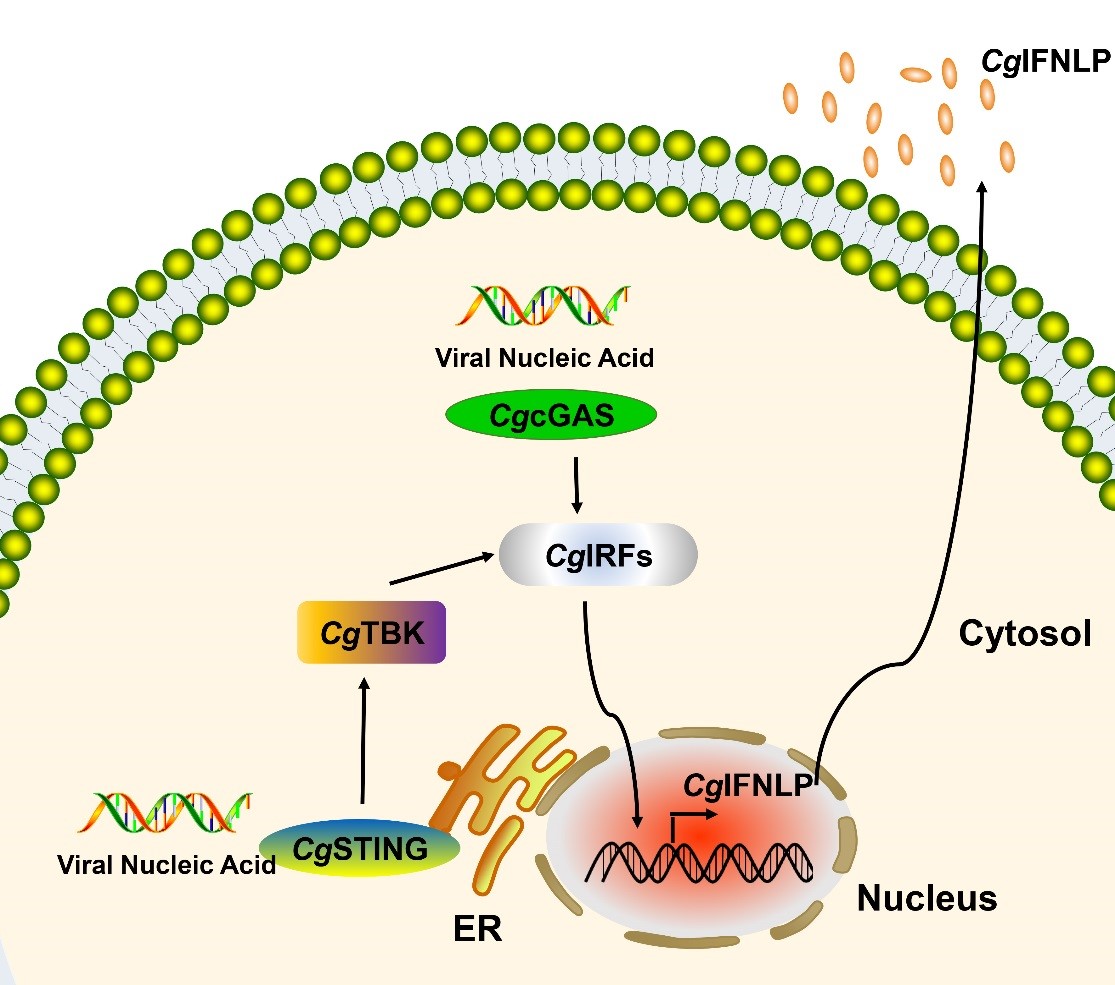


**Supplementary Figure 3. The cGAS/STING-TBKTBK1-IRF Regulatory Axis mediated *Cg*IFNLP expression.** Upon recognizing Double-stranded nucleic acid, the *Cg*cGAS and *Cg*STING synergistically facilitated the *Cg*IRFs-mediated *Cg*IFNLP production, which further induced the expression of *Cg*ISGs.

**Supplementary Table**

**Supplementary Table 1. Sequences of the primers used in this study**

|  | | **Primer** | | | | | | | Sequence (5’-3’) | |
| --- | --- | --- | --- | --- | --- | --- | --- | --- | --- | --- |
|  | | **Clone primers** | | | | | |  | | |
| P1 | | *Cg*cGAS-F | | | | | | CACCAAGGTAGCGCAAATGG | | |
| P2 | | *Cg*cGAS-R | | | | | | AACTTGAACATATTGTGGTTTGGA | | |
| P3 | | *Cg*STING-F | | | | | | CGAGCTC ATGGACGACTTTAGGAAC | | |
| P4 | | *Cg*STING-R | | | | | | CCGCTCGAG GCTTTTTAGAGAACACCG | | |
| P5 | | *Cg*IRF-8-F | | | | | | ATGGCAACAGAAATTGATATTCGC | | |
| P6 | | *Cg*IRF-8-R | | | | | | TCAGGTTTCCATTTGTCCATGG | | |
|  | **RT-PCR primers** | | |  | | | | | | |
| P7 | | | *Cg*cGAS-RT-F | | | CCGTTCAAAGATGGTGCAGG | | | | |
| P8 | | | *Cg*cGAS-RT-R | | | TTCCCTCGGACGTACAATGC | | | | |
| P9 | | | *Cg*STING- RT-F | | | CGGTCTTACGTCATCCCCAC | | | | |
| P10 | | | *Cg*STING- RT-R | | | CGTTGTTACGTTGGTGCGAG | | | | |
| P11 | | | *Cg*IFNLP-RT-F | | | CTTATTGCACTCCAACCTTCCA | | | | |
| P12 | | | *Cg*IFNLP-RT-R | | | AGACTGGGAGGATGTCCTGGAG | | | | |
| P13 | | | *Cg*IRF-1-RT-F | | | ATTCGTGTTTTCACATTCCCTACTC | | | | |
| P14 | | | *Cg*IRF-1-RT-R | | | ATTTCCACCTGGTCCTCCTTATC | | | | |
| P15 | | | *Cg*IRF-8-RT-F | | | CCGTATGGATGTCGGCTTTTC | | | | |
| P16 | | | *Cg*IRF-8-RT-R | | | AAAGCAGCCCCCTGTCTAACG | | | | |
| P17 | | | *Cg*TBK-RT-F | | | GGAGATCAGTGAAGTCATCGACA | | | | |
| P18 | | | *Cg*TBK-RT-R | | | GATTGGTGGTATTTCCGGCT | | | | |
| P19 | | | *Cg*Mx1-RT-F | | | AGACAGAGTAACGGGCCACA | | | | |
| P20 | | | *Cg*Mx1-RT-R | | | CAAGATCTGTGTTGGCGGGG | | | | |
| P21 | | | *Cg*Viperin-RT-F | | | TCAATTTTGCTGGTGGCGAG | | | | |
| P22 | | | *Cg*Viperin-RT-R | | | GGTTGGTTTCCGGGTCAAAG | | | | |
| P23 | | | *Cg*IF44-RT-F | | | TTTCTCTGAATGGCTCTGTCAA | | | | |
| P24 | | | *Cg*IF44-RT-R | | | GCCGTCTCTGCATTAAACTCT | | | | |
| P25 | | | EF-RT-F | | | AGTCACCAAGGCTGCACAGAAAG | | | | |
| P26 | | | EF-RT-R | | | TCCGACGTATTTCTTTGCGATGT | | | | |
|  | **Recombinant expression** | | | | | | | | |  |
| P27 | | *Cg*cGAS-Ex-F | | | CGCGGATCCCACCAAGGTAGCGCAAAT | | | | | |
| P28 | | *Cg*cGAS-Ex-R | | | CCGCTCGAGTTGTAAGAGACCATCTAGTTC | | | | | |
| P29 | | *Cg*STING-Ex-F | | | CGAGCTC ATGGACGACTTTAGGAAC | | | | | |
| P30 | | *Cg*STING-Ex-R | | | CCGCTCGAG GCTTTTTAGAGAACACCG | | | | | |
| P31 | | *Cg*IRF-8-Ex-F | | | GGAATTCCATATGATGGCAACAGAAATTGATATTCG | | | | | |
| P32 | | *Cg*IRF-8-Ex-R | | | CGCGGATCC GGTTTCCATTTGTCCATGG | | | | | |
|  | **RNA interference** | | | | | |  | | | |
| P33 | | *Cg*cGAS-RNAi-F | | | | GCGTAATACGACTCACTATAGGAGACAAGGGAGGAGAACCCA | | | | |
| P34 | | *Cg*cGAS-RNAi-R | | | | GCGTAATACGACTCACTATAGGTCGCGAGCTGCTTTATACTCT | | | | |
| P35 | | *Cg*STING-RNAi-F | | | | GCGTAATACGACTCACTATAGGGAACAACGCAGAAGGAAGGATG | | | | |
| P36 | | *Cg*STING-RNAi-R | | | | GCGTAATACGACTCACTATAGGTCGGGTGAACTAATGGACGG | | | | |
| P37 | | *Cg*IFNLP-RNAi-F | | | | TAATACGACTCACTATAGGGATGGAGAGGAAAAAGGATAAA | | | | |
| P38 | | *Cg*IFNLP-RNAi-R | | | | TAATACGACTCACTATAGGGTGTTTCTCTTTTCTGTGCTGT | | | | |
| P39 | | *Cg*TBK-RNAi-R | | | | GCGTAATACGACTCACTATAGGAGCAATGCCTTTGGTCTCCC | | | | |
| P40 | | *Cg*TBK-RNAi-F | | | | GCGTAATACGACTCACTATAGGTGGGCAACTCTTTGGACCAG | | | | |
| P41 | | EGFP- RNAi-F | | | | GCGTAATACGACTCACTATAGGTGGTCCCAATTCTCGTGGAAC | | | | |
| P42 | | EGFP-RNAi-R | | | | GCGTAATACGACTCACTATAGGCTTGAAGTTGACCTTGATGCC | | | | |
