# Supplementary figures and images for: The cGAS/STING–TBK1–IRF Regulatory Axis Orchestrates a Primitive Interferon-Like Antiviral Mechanism in Oyster
Source: Front Immunol. 2021 Jun 8;12:689783. doi: 10.3389/fimmu.2021.689783 (PMC8218723; doi:10.3389/fimmu.2021.689783)

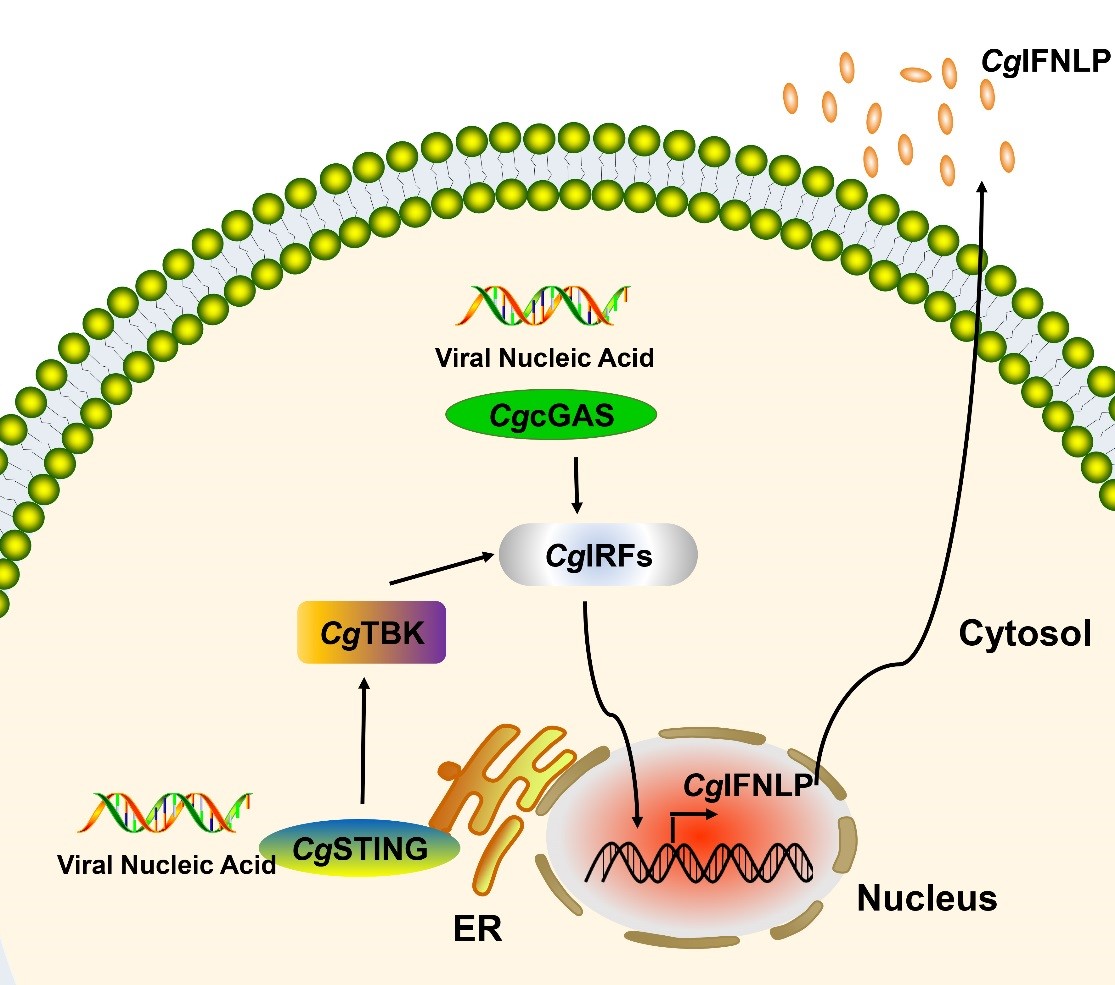

Supplement: Supplementary file 2 [file Image_1.jpeg]
